# Coulrophobia: An investigation of clinical features

**DOI:** 10.4102/sajpsychiatry.v28i0.1653

**Published:** 2022-01-19

**Authors:** Talia Planting, Sheri-Michelle Koopowitz, Dan J. Stein

**Affiliations:** 1 Department of Psychiatry and Neuroscience Institute, Faculty of Health Sciences, University of Cape Town, Cape Town, South Africa; 2 Unit on Risk and Resilience in Mental Disorders, South African Medical Research Council, Cape Town, South Africa

**Keywords:** Coulrophobia, phobia, specific, fear, survey, clowns

## Abstract

**Background:**

Coulrophobia refers to fear or disgust elicited by clowns, or images of clowns, and may be accompanied by significant distress. The medical literature on sociodemographic and clinical features of coulrophobia is, however, sparse.

**Aim:**

This study aimed to investigate coulrophobia sociodemographic and clinical features in an online support group.

**Setting:**

A self-administered questionnaire was distributed to an online support group for coulrophobia.

**Methods:**

Members of the online coulrophobia support group received a link to a self-administered questionnaire. The questionnaire focused on sociodemographic and clinical features, including fear-related and disgust-related symptoms, and included DSM-5 diagnostic criteria for specific phobia.

**Results:**

Of the 95 survey respondents, 79 were female respondents (mean age: 39.8 ± 12.6 years), with the mean age of onset 9 ± 6.1 years. Coulrophobia symptoms were associated with significant psychological distress and with impaired social functioning. About 7.4% of respondents reported severe anxiety with panic attacks. Comorbid disorders included major depressive disorder (9.5%), obsessive-compulsive disorder (5.3%) and panic disorder (3.2%). Individuals with fear-related symptoms were more likely to fulfil DSM-5 criteria for specific phobia.

**Conclusion:**

Coulrophobia is a phenomenon that warrants clinical attention, given its association with significant comorbidity, psychological distress and impaired functioning. Several sociodemographic and clinical features are consistent with a diagnosis of specific phobia, although future work employing clinician-administered diagnostic tools is needed to consolidate and extend the findings here.

## Introduction

Coulrophobia refers to significant distress, fear and/or revulsion when exposed to clowns.^1^ Those who suffer from this condition may record a longstanding fear and/or disgust associated with clowns, along with physical symptoms of distress and a need to escape when encountering them.^2,3^ It has been speculated that fear of clowns is linked to the socially unacceptable qualities that clowns embody; for example, the emphasis in popular culture on the dark side of clowns, or the use of clown costumes by certain criminal groups and murderers.^2^ Similarly, it has been pointed out that clowns may be perceived as threatening because of their subversive, deviant and, at times, violent behaviour, and this, coupled with the combination of exaggerated features that give the sense of both real and uncanny qualities, makes them particularly threatening for certain individuals.^4^

Several online support groups have emerged for those individuals who share similar negative experiences of clowns. This includes the popular social media site, Facebook, with over 8000 individuals identifying having either a fear or revulsion of clowns on a particular support group webpage. These support groups may include links to articles, pictures and videos depicting clowns as portrayed by authors, films or criminals. Posted comments may include references to longstanding fear, disgust and mistrust of clowns; physical symptoms of distress (such as emesis); and the need to escape when encountering clowns.

Although coulrophobia has been discussed in scholarly papers in the humanities, it is not well-documented in the medical literature. One of the studies examined the prevalence of coulrophobia in hospitalised children because of the use of medical clowns; of 1160 hospitalised children, 14 children demonstrated coulrophobia, of which 12 were female.^3^ However, the medical literature examining the sociodemographic and clinical features of coulrophobia in adults is scant.

The DSM-5 specific phobia subtype of ‘other’ includes ‘costumed characters’.^5^ There are, however, many gaps in our knowledge of coulrophobia. Firstly, although specific phobia begins early in life and is more common in women, these characteristics have not been well-documented in coulrophobia.^3^ Secondly, in specific phobia there are multiple comorbidities, and there may be panic attacks, which have only rarely been noted in coulrophobia.^2^ Thirdly, in specific phobia there are elements of fear and disgust.^6^ Although such elements are apparent in some writing on clowns, the medical literature has not explored this distinction in coulrophobia.

This study aims to expand the knowledge of a phenomenon that is underreported in the scientific literature, despite its apparent occurrence in thousands of individuals. We addressed the sociodemographic correlates of coulrophobia (age and sex) and its clinical features (e.g. age of onset, distress, impairment, and comorbidity, fear-related and disgust-related symptoms).

## Methods

### Sample

A survey of adults (≥ 18 years) who self-identified as having or having had a fear or disgust of clowns and were part of an online support group for those with coulrophobia was conducted. There were approximately 8000 members in this group and 95 respondents completed the questionnaires, resulting in a response rate of approximately 1.2%.

### Questionnaires

A self-administered online questionnaire of 57 questions was developed to determine the sociodemographic and clinical features of coulrophobia in an online support group. Sociodemographic questions included age, sex and highest level of education. Clinical features included age of onset, symptom duration, extent of psychological distress and functional impairment, precipitants and family history of coulrophobia. Participants were asked if they had received a psychiatric diagnosis to ascertain comorbidities. The questionnaire included measures of distress and impairment (the Kessler Psychological Distress Scale (K10).^7^ and the Sheehan Disability Scale.^8^ The Kessler Psychological Distress Scale is a 10-item scale that measures non-specific psychological distress in clinical and non-clinical samples.^7^ The Sheehan Disability Scale measures impairment and disability in work, social, and family life in community and clinic samples.^8^

The DSM-5 diagnostic criteria for specific phobia were included in the survey. Questions such as ‘[d]o you have fear of, disgust toward or anxiety about clowns?’ and ‘[d]o you actively try avoid clowns?’ were included, as well as ‘[d]oes your fear, disgust, anxiety or avoidance of clowns make you very upset or does it interfere with important areas of your daily life (e.g. your work, social life or relationships)?’. Additionally, questions that addressed whether symptoms were mainly fear-related or mainly disgust-related were included. For example, ‘[*w*]hen looking at an image of clowns, which of the following describes your experience the best?’ with multiple choice answers such as ‘I experience only fear’, or ‘I experience only disgust/ revulsion’, or ‘I experience mostly fear, but also disgust/revulsion’, ‘I experience mostly disgust/ revulsion, but also fear’, or ‘I experience the same amount of fear and disgust/ revulsion’.

An explanation of the study was provided in a post on the online support group, with a link to ‘SurveyMonkey’ for the survey, which took approximately 20 min to complete.

### Statistical analysis

In order to assess the sociodemographic and clinical correlates of coulrophobia, frequencies of categorical variables, and means and standard deviations of continuous variables in the questionnaire were calculated. Those with only fear-related coulrophobia symptoms and those with only disgust-related coulrophobia symptoms were compared in terms of sociodemographic and clinical features using Chi-square tests and *t*-tests, as appropriate, with significance set at α = 0.05.

### Ethical considerations

The University of Cape Town, Faculty of Health Sciences Human Research Ethics Committee approved the study. All participants were informed of their anonymity, and that their participation was voluntary. All participants provided informed consent before completing the survey.

## Results

### Sociodemographic features

A total of 95 respondents were included in the analyses. A greater proportion of respondents were female (83.2%) than male respondents (16.8%), with an age range of 19–65 years (mean = 39.8 ± 12.6). Just over half (54%) of the respondents were married or in a domestic partnership, whilst 29% were single and never married. Tertiary-level education (university or college level) had been completed by 59% of respondents, and high school education by 17% of respondents. Fifty-nine per cent were employed full time, 8% were retired, 6% were studying and 6% were homemakers.

### Clinical features

The age of symptom onset ranged from 1 to 35 years (mean = 9 ± 6.12). The duration of symptoms ranged from 6 to 60 years (mean = 30.4 ± 12.9). The majority of respondents (89.5%) noted no remission of symptoms from the initial onset, with only 10.5% having a period of remission since the symptom onset. About 3.2% of those experiencing a remission had a 10-year decrease in symptoms. Respondents spent an average of 1.3 h (SD = 3.5) per week thinking about clowns. About 3.2% of respondents experience coulrophobia once per day, 21.1% once per week, 24.2% once per month, 45.3% once per year and 5.3% less than once per year.

Regarding anxiety and self-reported panic attacks, 7.4% of respondents reported experiencing severe anxiety with self-reported panic attacks from exposure to clowns. Furthermore, 7.4% of respondents experienced severe anxiety without panic attacks, 29.5% experienced moderate anxiety, 46.3% experienced mild anxiety and 9.5% reported experiencing no anxiety. Of the respondents reporting self-reported panic attacks related to their coulrophobia, 8.4% had one to two panic attacks per month, whilst 1.1% reported more than 10 panic attacks per month. When exposed to clowns, 43.9% of participants experienced palpitations, 27% experienced shortness of breath and 14.2% felt nauseous. A total of 14.9% of respondents identified feeling humiliated or ashamed of their coulrophobia.

A total of 62.1% of participants identified experiencing minimal psychological distress, 13.7% experiencing mild distress, 9.5% moderate distress and 14.7% severe psychological distress by their coulrophobia. Participants reported severe disruption of work or school functioning (6.3%), of social functioning (13.7%) and of family life (9.5%) because of their coulrophobia.

Diagnoses reported included major depressive disorder (9.5%), bipolar mood disorder (2%), obsessive-compulsive disorder (5.3%), panic disorder (3.2%), social anxiety disorder (3.2%) and specific phobia other than coulrophobia (1%). The majority of respondents (72.6%) had not, however, been diagnosed for a psychiatric disorder by a healthcare provider.

The majority of participants had not received any treatment for their coulrophobia (96.8%), whilst 1.1% had received counselling and 2.2% reported receiving other forms of treatment. Of those who received treatment for their coulrophobia, none identified it as helpful.

### Questions related to specific phobia

A total of 9.5% of respondents currently fulfilled DSM-5 criteria for a specific phobia involving clowns, and 42.1% currently fulfilled DSM-5 criteria for a specific phobia excluding the distress or impairment criterion.

Many respondents (69.5%) did not identify a specific experience triggering their coulrophobia. Of the 30.5% who identified a specific trigger, 7.4% identified the film ‘IT’ as the precipitant, whilst 3% identified a clown at the circus, and 3% identified a clown in their personal space. Three-quarters of all respondents (75.8%) experienced symptoms only on seeing a clown, whilst almost a quarter of respondents (21.1%) experienced symptoms at least once per week. Furthermore, 22.1% of respondents identified having a relative with coulrophobia.

Almost 18% of respondents reported only fear associated with clowns, whereas 27.4% experienced not only fear but also disgust. Additionally, 15.8% experienced only disgust associated with clowns and 22.1% experienced mostly disgust but some fear. Approximately 17% of respondents experienced equal amounts of fear and disgust on exposure to clowns.

Comparison of the only fear-related versus the only disgust-related group found that the only fear-related group had a greater proportion of female respondents (94.1% vs. 60%; *p* = 0.02), greater likelihood of shortness of breath (76.5% vs. 13.3%), nausea (35.3% vs. 13.3%) and palpitations (82.4% vs. 13.3%), and were more likely to meet the DSM-5 criteria for specific phobia (17.6% vs. 6.7%).

## Discussion

The major findings of this study were (1) coulrophobia has an early age of onset, which is more common in female participants, and may be accompanied by significant distress, impairment and comorbidity; (2) some participants with fear-only symptoms were more likely to meet DSM-5 criteria than those with disgust-only symptoms, reflecting lower distress or impairment in the former group.

Notably, several features of coulrophobia are redolent of specific phobia, including early age of onset, female predominance, chronic duration and the presence of both fear-related and disgust-related symptoms. The data that coulrophobia has an early age of onset is more common in female participants and may be accompanied by significant distress, impairment and comorbidity are also consistent with the few reports of coulrophobia in the medical literature. For example, in a sample of hospitalised children,^3^ the majority of children with coulrophobia were female children. Further, given the presence of comorbid disorders, these should be carefully screened for in individuals who present with coulrophobia.

The view that coulrophobia is best conceptualised as a specific phobia is strengthened by the finding that many individuals in our sample meet the current DSM-5 criteria for this disorder, and particularly so if they experienced fear-related symptoms. A larger number meet such criteria, if the clinical criterion of distress or impairment is waived; this suggests that many of our respondents have suffered from a sub-clinical condition or have improved over time. Further work using clinician-rated diagnostic instruments is needed to determine whether individuals with current coulrophobia meet DSM-5 criteria for specific phobia and respond to evidence-based treatments for specific phobia.

A number of limitations of this study deserve emphasis. Firstly, members of this support group may not be representative of those with coulrophobia, and the response rate was low. Nevertheless, it has been suggested that an online population is likely to be more representative as it accesses an international group of people with a broader demographic distribution.^9^ Secondly, all data were based on self-report with no clinical validation. Comorbidity estimates are based on diagnoses received from a healthcare provider and are therefore likely to be conservative. Nevertheless, the anonymity and voluntary nature of survey completion are likely to have facilitated valid responses.^9^ Thirdly, this questionnaire was derived from the literature on specific phobia and provided relatively little information on reasons for why people might not have met the DSM-5 criteria for this condition. Comprehensive clinical assessments would be useful to address this point in future work.

## Conclusion

Coulrophobia is a phenomenon that warrants clinical attention, given its association with significant comorbidity, psychological distress and impaired functioning. Several clinical features, including early age of onset and female preponderance, are similar to those of specific phobia, and some individuals with coulrophobia may be usefully conceptualised as suffering from this condition. Further research is needed, however, to consolidate these observations and recommendations.
